# Supplementary material for: Legumes of the Sardinia Island: Knowledge on Symbiotic and Endophytic Bacteria and Interactive Software Tool for Plant Species Determination
Source: Plants (Basel). 2022 Jun 6;11(11):1521. doi: 10.3390/plants11111521 (PMC9183093; doi:10.3390/plants11111521)
Supplement: Supplementary file 1 [file plants-11-01521-s001.zip › Document S1. English version Instructions for Botabase Keys.doc]

**B O T A B A S E	K E YS**

**B o t a n i c D a t a b a s e w i t h B u i l t–I n	K e y s**

**Computer Application for Plant Species Determination**

**based on the Subtractive Keys Principle and operating in Microsoft Excel**

**Idea and Construction: Andrea Squartini, 2004**

**Version: Leguminosae of Sardinia**

**Note:** the version of the attached file relates to data based on the first version of Flora d’Italia but the additional species can be entered by adding corresponding rows and data in the appropriate columns.

The reference Excel file is in .xls format and can also be used with Excel versions 1997-2003. The instructions indicate both the choices for using this version and those for the advanced versions of Excel (.xlsx)

**Walkthrough demonstration and use of the filter**

Open the Excel file “English version Botabase Keys for Legumes of Sardinia Island.xls”, containing the data for all the 189 species of Leguminosae found in the area of choice. The data have been entered from the official national botanical guide (Pignatti, 1982, Flora d’Italia, volume I). Names (and synonyms in racket are in column A.

If the Excel filter function is not already selected, you can open it from the menu: Data 🡪Filter 🡪 Autofilter

On top of each column you have now the pull-down menus from which you will be able to select the criteria for filtration (*If you want* *to undo the filter: Data 🡪Filter 🡪 Show all*).

**Form** (column B) This is the plant life form

A : Annual, B : Biennial, P : Perennial, PS : Perennial Shrub (woody), PT, Perennial Tree (woody with trunk)

If for example the specimen we are dealing with is a woody plant we can exclude at once all the herbaceous ones as follows: from the filter pull-down we choose:

Text filter > Custom filter (Excel .xlsx versions) or > Custom... (Excel 1997-2003)

Show rows where :

form

*equals* PS

or

*equals* PS, PT

(Notice that now, the taskbar tells us that our list has been purged reducing the number from 189 down to 26 species left!)

If our specimen instead happens to be an herbaceous plant we want to exclude all the woody ones:

Text filter > Custom filter (Excel .xlsx versions) or > Custom... (Excel 1997-2003)

Show rows where :

form

*does not equal* PS

and

*does not equal* PS, PT

Note 1: it is important in the first example (looking for a woody plant) in which the criteria are: “equals”, that the operator be “OR” (not “AND” !) otherwise the program will look for cells in which it finds both PS and PS,PT, repeated in the same cell but naturally such cells do not exist.

Instead, in the second example, looking for an (herbaceous plant), in which the criteria are “does not equal” the operator must be “AND”(not “OR”). In fact when we say “does not equal PS” we exclude PS, but if we were to add “or does not equal PT” this would re-include PS and as a consequence we would not discard any of the two.

**Minimum altitude** (Column C)

Suppose we collected our specimen on a site located at an altitude of about 137 meters above sea level, (the value can be easily read either on charts, or via GPS on the spot). It is not strictly necessary to attain particular accuracy an approximation is perfectly suitable to the method let’s suppose we are able to tell that our altitude was between 100 and 150 m a.s.l..

With the filter on Column C we want to exclude all the plants whose minimal altitude is superior to the level at which we found ours (e.g. all the mountain plants that will never occur below a certain altitude). We proceed as follows:

Text filter > Custom filter (Excel .xlsx versions) or > Custom... (Excel 1997-2003)

Show rows where :

min. altitude (m)

*is less than* 150

Note 2: let us introduce here a major feature of this method, i.e. the **issue of Forgiveness-Flexibility** that highly reduces the loss of true positives. Even though theoretically when we choose the value of 150, this should exclude the plants that can not be our specimen, that is all those that never grow below 150 m, in reality in biology behaviour and forms tend to be rather elastic. Therefore, it is better for this and for all the other filtrations of traits that vary in a quantitative continuous manner (height, time of flowering, flower or leaf dimensions, number of leaflets etc., “**to keep ourselves a bit looser”** and to be on the safe side we should not write 150 but at least 300 o 400 m . In fact, no matter how accurate and updated are our sources of data, it is possible that the plant of choice, reported as never found at altitudes lower than a certain value, could occasionally occur below those. If that were the case, if we stick too close to our measured boundary, we could have filtered it off from the list of the plants left.

Therefore it is better to enter:

Text filter > Custom filter (Excel .xlsx versions) or > Custom... (Excel 1997-2003)

Show rows where :

min. altitude (m)

*is less than* 300

**Maximum altitude** (Column D)

Here we want to get rid of all the species that will never be pushed at an altitude like the one where we collected our plant. In the case of our example as the altitude is moderate we will basically eliminate a series of littoral species which are bound to the shores and so on. Instead if we had found ours in a high mountain belt, the operation would dramatically skim our list making all the non-mountain plants disappear. The filter is to be used as follows:

Text filter > Custom filter (Excel .xlsx versions) or > Custom... (Excel 1997-2003)

Show rows where :

max. altitude (m)

*is greater than* 50

As we recommended in Note 2 we kept ourselves loose (in this case in the direction below).

Note 3. In order to better memorize how to set the filters on traits of minima and maxima it is helpful to keep in mind the following rule of the thumb :

When a column is concerned with *minima* *,*you must set “*is less than”*, and then write a value that is sufficiently *greater* than the one we observed for our specimen .

When a column is instead concerned with *maxima* you must set “*is greater than”*, and then write a value that is sufficiently *smaller* than the one we observed for our specimen .

Therefore, for columns of minima such as “minimum altitude”, “minimum height”, “flowers starting from the month of ..” etc. remember the mnemonic sequence “***minima-less than-greater***”

While for columns of maxima as “maximum altitude”, “maximum height”, “flowers until the month of ..” etc. remember the mnemonic sequence “***maxima-greater than-smaller***”

**Minimum height** (Column E)

Example, the plant is 40 cm tall, we keep in mind the law of staying somewhat looser explained in Note 2

Text filter > Custom filter (Excel .xlsx versions) or > Custom... (Excel 1997-2003)

Show rows where :

min height (cm)

*is less than* 60

(It is a Column of *minima*, the criterium is therefore “*is less than”*, and we write a value which *is greater than the one observed*, that is, having observed 40 cm we write at least 60.

**Maximum height** (Column F)

Text filter > Custom filter (Excel .xlsx versions) or > Custom... (Excel 1997-2003)

Show rows where :

max. height (cm)

*is greater than* 20

(It is a Column of *maxima*, the criterium is therefore “*is greater than”*, and we write a value which *is smaller than the one observed*. That is, having observed 40 cm we write a value like 20.

**Flowers since:**  (Column G)

The numbers are those of the months (3= March, 4=April etc.)

Suppose we found our plant, in bloom, on may 7th , we want to filter off those plants that will bloom in later times. Again complying to the elasticity rule discussed in Note 2, it is better not to exclude abruptly all those that are known to flower from June, it is wiser to stay a month looser and exclude those that are declared as flowering from July onwards. It is in fact important to consider the possible anticipations or retards of the season and climate fluctuations that every year can induce some shifts from phenology rules. Therefore :

Text filter > Custom filter (Excel .xlsx versions) or > Custom... (Excel 1997-2003)

Show rows where :

fiowers since (month)

*is less than* 7

**Flowers until :**  (Column H)

Here we want to exclude all those that in May would surely be past their flowering time. As above, rather than setting the cut off in April we put March:

Text filter > Custom filter (Excel .xlsx versions) or > Custom... (Excel 1997-2003)

Show rows where :

fiowers until (month)

*is greater than* 3

**Leaves** (Column I)

In this column the variable traits are not quantitative but qualitative; we will not use operators for comparison of the ‘lesser’ or ‘greater’ type, but rather we will look for *words* corresponding to the observed type. The function of text search in the filter enables to find words or parts of them, including small portions, Therefore if we have leaves with three leaflets (trifoliolate) we could just set the filter to look for *trif* , or even for *oliol* etc. This makes the search very versatile and allows the use of very complex descriptions in the cells.

Let’s begin for example by checking if our plant has leaves with tendrils, we just set the filter :

Text filter > Custom filter (Excel .xlsx versions) or > Custom... (Excel 1997-2003)

Show rows where :

leaves

*contains*	*tendr*

if moreover we notice that our specimen has also a winged stem (flattened longitudinal membranes along the stem) we can better set:

Text filter > Custom filter (Excel .xlsx versions) or > Custom... (Excel 1997-2003)

Show rows where :

leaves

*contains*	*tendr*

AND

*contains* *wing*

If instead the plant has winged stem but no tendrils we set :

Text filter > Custom filter (Excel .xlsx versions) or > Custom... (Excel 1997-2003)

Show rows where :

leaves

*contains* *wing*

AND

*does not contain*	*tendr*

(In this case we would have already reached the species determination as the only one with winged stem and no tendrils is *Lathyrus nissolia*)

If instead, as the majority of plants, ours has neither tendril nor winged stem we observe the leaves.

A first type easy to recognize is the case of the palmate leaves, they are made by leaflets but all are attached in the same point. If such leaves are present we select:

*contains* **palmate**.

If leaves are not palmate, they can be simple or made by a number of leaflets (pinnate). The number of leaflets in Leguminosae is variable, and there can be a terminal single one or a terminal pair. The number can be odd or even . To choose how to filter you need to count leaflets. The numbers in the following list correspond to the number of leaflets. The word **in bold** is the term to enter in the filter function after the operator *contains* :

1) **Simple** : a leaf that is not made by leaflets (entire)
2) **1pinnate** (leaf with a single pair, has a total of 2 leaflets)
3) **trifoliolate :** leaf with three leaflets (as e.g. in clover)

3bis) **trifoliolate, central leaflet petiolulate** (the three leaves are not attached in the same point, the central one has its own petiole)

4) **2pinnate** (two pairs of leaflets, i.e. 4 leaflets)
5) **5**	( five leaflets “lizard shape”)

5bis) **3+2** (still five but the basal pair is visibly different being enlarged stipules)

>5) **>5foliolate (**when the leaflets are more than five and the leaf has 6 or 6+1leaflets or quadri-pinnate (8 or 8+1 leaflets) etc. etc.

Inn case the plant contains more different kinds of the above described leaves, it will be sufficient to enter *contains* ***mix***

If the leaves do not belong to any of the well defined types it will be sufficient to enter

*contains* **particular**

If for example you are not sure whether the central leaflet is sessile or petiolulate (as the petiole could be very short ) you can just enter *contains* *trif*. This does not exclude the petiolulate cases and includes all the trifoliolate.

Example

The plant, with no tendrils nor winged stem, has 12 pairs of leaflets

Text filter > Custom filter (Excel .xlsx versions) or > Custom... (Excel 1997-2003)

Show rows where :

leaves

*contains ****>5foli***

AND

*does not contain*	*tendr*

Note 4. The terms of text have to be written exactly as in the cells **1pinnate** (etc.) without space between 1 and pinnate otherwise that text will not be found.

**Minimum number of leaflets** (Column J)

In case we chose the leaves with many leaflets (>5foliolate) or those with tendrils, we have the chance to use also the two columns J and K, in which we can set exact values by counting the leaflets (if instead we had chosen leaves of the other types the count was already known and we can skip to the further columns).

If for example our plant has leaves with 16 leaflets

Text filter > Custom filter (Excel .xlsx versions) or > Custom... (Excel 1997-2003)

Show rows where :

n.min leaflets

*is less than* 18

(again for the rule of the thumb it is wiser to say 18 instead of 16)

**Maximum number of leaflets** (Column K)

Still based on the example with 16 leaflets

Text filter > Custom filter (Excel .xlsx versions) or > Custom... (Excel 1997-2003)

Show rows where :

n.max leaflets

*is greater than* 14

**Leaflet shape** (o or leaf shape for species with entire leaves) (Column L)

This is a descriptive column, not intended for filtration but useful to verify results at this stage.

IMPORTANT : in the four columns on leaflet width or length not all the cells contain data as for some species the botanical textbooks do not give values. This is not a problem provided that one adopts the simple additional practice of selecting, besides the dimensional criterium of choice, ALSO THE EMPTY CELLS. It will be sufficient to insert also “OR” *equals*	leaving blank the second windows of choice. This means “*equals* empty cells” .

Suppose we have a plant whose leaflets (upon measuring a fair number and on completely mature and developed leaves) in width are never smaller than 3 mm and never larger than 6 mm, and in length are never shorther than 10 mm and never longer than 13 mm.

**Leaflets minimum width** (or leaves, for species with entire leaves) (Column M)

Text filter > Custom filter (Excel .xlsx versions) or > Custom... (Excel 1997-2003)

Show rows where :

leaflets min. width (mm)

*is less than* 5

OR

*equals*

Important : use OR (not AND) and leave blank the field after *equals*, only this way you will not discard the empty cells.

**Leaflets maximum width** (or leaves, for species with entire leaves) (Column N)

Text filter > Custom filter (Excel .xlsx versions) or > Custom... (Excel 1997-2003)

Show rows where :

leaflets max. width (mm)

*is greater than* 4

OR

*equals*

(blank field after *equals)*

**Leaflets minimum length** (or leaves, for species with entire leaves) (Column O)

Text filter > Custom filter (Excel .xlsx versions) or > Custom... (Excel 1997-2003)

Show rows where :

leaflets min. length (mm)

*is less than* 12

OR

*equals*

**Leaflets maximum length** (or leaves, for species with entire leaves) (Column P)

Text filter > Custom filter (Excel .xlsx versions) or > Custom... (Excel 1997-2003)

Show rows where :

leaflets max. length (mm)

*is greater than* 11

OR

*equals*

It is critical to remember to write also equals and leave the blank otherwise, by filtering away rows with empty cells we risk to exclude plants that could be ours but for which the minima and maxima were not known to the guide. As additional reminder for this the headings of these columns are written in red.

**Inflorescence** (Column Q) The way flowers are arranged

Distinctions to made, first, observe if there are isolated flowers (single), in that case:

Text filter > Custom filter (Excel .xlsx versions) or > Custom... (Excel 1997-2003)

Show rows where :

inflorescence

*contains* *singl*

AND

*contains* *1-*

This way we select all cases in which flowers are isolated or those in which you can have both single isolated flowers and non-isolated flowers.

If you do not observe any isolated flower as they are grouped you will work on the inflorescence type. There are three basic possibilities.: **racemes** (“grape-like”) in which there is a more or less elongated axis on which flowers are inserted, (at times numerous but they could also be as few as two only); **heads (“**pon pon-like”**)** in which flowers are inserted around a same point on top of an axis. (Even when the flowers inserted this way were just 2 this will still be technically a head as long as they are inserted at the same point, while in the raceme they will be inserted in different points along the axis.) ; **fascicles**, in which flowers, in groups (or even as a single flower for groups of 1 ) are not borne in an inflorescence with axis, but directly on the stem in a sessile or peduncled way.

Therefore, in the case of racemes :

Text filter > Custom filter (Excel .xlsx versions) or > Custom... (Excel 1997-2003)

Show rows where :

inflorescence

*contains* *racem*

while in case of heads

Text filter > Custom filter (Excel .xlsx versions) or > Custom... (Excel 1997-2003)

Show rows where :

inflorescence

*contains* *head*

while if the case is that of fascicles or isolated flowers (sessile or peduncled)

Text filter > Custom filter (Excel .xlsx versions) or > Custom... (Excel 1997-2003)

Show rows where :

inflorescence

*contains* *sess*

AND

*contains* *fasc*

**Minimum number of flowers per inflorescence** (Column R)

Even though not all cells of this column and of the next, contain data as the exact number can be missing in literature, this does not represent a problem for the filtration process as, like the case of columns M,N,O,P, it will be sufficient to add the empty cells to the filter criterium.

Imagine we counted the flowers in various inflorescences of our plant and that the minimum be 12 and the maximum 20.

Text filter > Custom filter (Excel .xlsx versions) or > Custom... (Excel 1997-2003)

Show rows where :

n.min flowers/inflorescence

*is less than* 15

OR

*equals*

(blank after equals)

**Maximum number of flowers per inflorescence** (Column S)

On the same example

Text filter > Custom filter (Excel .xlsx versions) or > Custom... (Excel 1997-2003)

Show rows where :

n.max flowers/inflorescence

*is greater than* 17

OR

*equals*

(blank after equals)

**Minimum size of flower head inflorescence** (Column T)

This column is to be used when you are left with species that have flowers borne by inflorescences of the head type (e.g. clovers and alike), the heads can be round or elongated (if they are round the minimum dimension will be equal or similar to the maximum dimension, while if they are elongated the minimum dimension will be the width). Suppose we have a head 15 mm wide and 30 mm long.

IMPORTANT : also for this column and for the following, not all the cells contain data as for some species the botanical textbooks do not give values. This is not a problem, as long as we select, besides the dimensional criterium of choice, ALSO THE EMPTY CELLS. It will be sufficient to insert also “OR” *equals*	leaving blank the second windows of choice. This means “*equals* empty cells” .

Text filter > Custom filter (Excel .xlsx versions) or > Custom... (Excel 1997-2003)

Show rows where :

min.size flowerhead (mm)

*is less than* 18

OR

*equals*

Important : again remember to use OR ( not AND) and leave the blank field after *equals*, not to discard cells where data are not available

**Maximum size of flower head inflorescence** (Column U)

Text filter > Custom filter (Excel .xlsx versions) or > Custom... (Excel 1997-2003)

Show rows where :

max. size flowerhead (mm)

*is greater than* 26

OR

*equals*

**Colour of corolla** (Column V)

Given the possible subjectivity and variability insert a part of the word to include the possible different nuances. For example if it is **red** you can set the filter not for *equals* **red** but rather for *contains* ***red*** that will therefore include all the derivate words like reddish, red-brown, etc. )

But do not write it any shorter than necessary (as ***re***) since **re** will include also colours as g**re**en.

Esempio :

Text filter > Custom filter (Excel .xlsx versions) or > Custom... (Excel 1997-2003)

Show rows where :

corolla colour

*contains* *red*

**Minimum flower size** (Column W)

Suppose that our corolla is 10 mm long and keep in mind the rule of thumb

Text filter > Custom filter (Excel .xlsx versions) or > Custom... (Excel 1997-2003)

Show rows where :

min. length corolla (mm)

*is less than* 12

**Maximum flower size** (Column X)

Text filter > Custom filter (Excel .xlsx versions) or > Custom... (Excel 1997-2003)

Show rows where :

max. length corolla (mm)

*is greater than* 8

**Other notes** (Column Y)

This is not necessarily a column to filter but can give useful accessory info to be read.

**Legume** (Column Z)

Strictly speaking also this column could be simply a way to verify information. Also in many cases at the time you have the flower you may not have yet the mature fruit. If instead you have the legume there are some clearly distinguishable types as those of *Medicago*, (Custom..., *contains* *spiral*), those articulate (with articles) of *Hedysarum* or *Coronilla* (*contains* *artic*), those with apparent holes of some *Hippocrepis* (*contains* *hol*), or those with wings of *Tetragonolobus* (*contains*  *wing*). For other features, do not filter but limit to the info as check up. Moreover, at this stage normally the determination could have already reached its completion (single plant left).

**Habitat** (Column AA)

Useful to verify the result. The use of the filter if still necessary is possible only if: (1) the cell refers to an univocal habitat and (2) our plant **has not** been found in a site belonging to that kind of habitat. In that case we can discard that habitat and its associated rows. Example, you found your plant on a field, you can thence discard those that live only in woods. For those plants whose habitat is only of a certain kind (univocal habitat) the cell has been highlighted with colour and brings in the text a code of three capital letters which are specific of that univocal habitat. The code words, to allow filtering, are devised in a way that they do not recur also in cells with non-univocal habitat. For example the word woods can occur also along with other words in non-univocal habitats but the code WDS is only in cells where the woods is the only habitat for that plant. The types of coded univocal habitats are: WDS : (forest and similar); MAR, (maritime, sea) ; DRY : (arid, dry environment, including garrigue; WET : humid areas; PEAT : heath land; CLF, cliffs.

Cells not highlighted in color contain more than one possible habitat as the plant of that row can be found in different environments and can not be excluded upon this principle.

To discard the plants of woods (if ours was found outside of the woods) we will set:

Text filter > Custom filter (Excel .xlsx versions) or > Custom... (Excel 1997-2003)

Show rows where :

habitat

*does not contain* WDS

**Habitat note** (Column AB)

Not for filtering but useful info for verification.

**Substrate** (Column AC) Indicates, when the term is univocal (example : **limestone,** example **quartz)** an exclusivity of the plant for that substrate. But obviously other plants whose cells have no indications of exclusivity could also be found in one of those particular substrates. The use of the filter here is possible only if: (1) the cell refers an univocal substrate and (2) our plant **has not** been found on that type of substrate. We can in that case discard the plants that thrive only on that. Example we found our specimen in a site that we know **is not** limestone .

Text filter > Custom filter (Excel .xlsx versions) or > Custom... (Excel 1997-2003)

Show rows where :

substrate

*does not equal* limestone

Note 5 here we must say *does not equal* limestone, (and not, *does not* *contain **limes* or similar internal portions of the word), because we do not want to discard rows where, for example, the substrate can be referred to as ‘generally limestone’ so the cell will contain the word limestone and its parts. We do not want to discard those plants that generally dwell on limestone but occasionally could be somewhere else.

**Abundance**(Column AD)

CC : extremely common, C : common, R : rare, RR : extremely rare

Column not for filtering, but useful as test and to judge also from this standpoint the likelihood that your identification result be correct.

**Endemics** (Column AE)

Column of mere information, not for filter, the signs here indicate endemics, whose restricted range includes also (x) or only (**X**) Sardinia.

**Geographical** **Notes** (Column AF)

Listed are data known for those species that in the area of this database are known to be limited to the indicated localities. (in the absence of indications a generalized distribution is intended as possible).

Example # 1, I found a plant in the surroundings of the town of Sassari. That means that it does not concern any of the particular different places listed in those cells that contain data. Therefore I can filter and discard the plants that would be found only in those places..

Text filter > Custom filter (Excel .xlsx versions) or > Custom... (Excel 1997-2003)

Show rows where :

Geographical notes (Sardinia)

*equals*

That is equals ‘blank’, simply not writing anything after equals (or choosing directly empty from the pull down window of the filter, without even choosing Custom…. This will leave only those rows where there is no geographical indication.

Example # 2. I found a plant in Cagliari

Text filter > Custom filter (Excel .xlsx versions) or > Custom... (Excel 1997-2003)

Show rows where :

Geographical notes (Sardinia)

*equals*

OR

*equals* *cagl.*

**DETERMINATION**

Having reached the end of the available subtractive keys, we will be ideally left with a single plant. If we still have more than one row, there are simple operations that will clear the doubt: :

1) Read the cells of the rows with which we are left, in particular the descriptive ones that were not used for filter: legume, leaf shapes, other notes etc. easily these can be very diagnostic at this stage.
2) Verify your exact diagnosis or compare the few alternatives that you have left by connecting through internet, opening Google, selecting the search engine Google Images and copying-pasting the plant botanical name you are checking in Google’s window. Nowadays the web has reached such a richness that virtually all plants have at least an image in it to be looked at. If you still had more than one possible candidate to name your specimen, the pictures will give you the answer at a glance.

**MEMO FOR OPERATIONS**

**Column B**	if herbaceous	*does not equal* PS	**and**	*does not equal* PS,PT

If woody	*equals* PS	**or**	*equals* PS,PT

**Columns C,E,G,J,W	(minima)**	*is less than* ... a value higher than ours

**Columns M,O,R,T**	**(minima)** *is less than* ... a value higher than ours

**or**	*equals ...	(leave blank)*

**Columns D,F,H,K,X**	(**maxima**)	*is greater than*	...a value smaller than ours

**Columns N,P,S,U**	(**maxima**)	*is greater than*	...a value smaller than ours

**or**	*equals ...	(leave blank)*

**Column I**	if it has leaves with tendrils	*contains*	*tendr*

Tendrils and winged stem	*contains*	*tendr*	and	*contains* *wing*

Winged stem and no tendrils *contains* *wing*	and	*does not contain*	*tendr*

No tendrils notr winged stem, choose one of these : *simpl*, *1pinna*,*trifoli*,*petiulat*, *2pinna*, *5pinna*,*3+2*, *>5foli*, *palma*, *mixed*, *particular*

*contains*  *(the chosen one)*	and	*does not contain*	*tendr*

**Column Q**	isolated flowers	*contains* *singl*	and	*contains* *1-*

No isolated flowers	choose among:

*contains* *racem*

*contains* *head*

*contains* *sess*	and	*contains* *fasc*

**Column V**	*contains* *(part of the color word, e.g. : “red”)*

**Column Z**	if available and one of the types *spiral*, *artic*, *fori* o *ali*

*contains* *(one of those)*

**Columns AA, AC**	if applicable discard a couple of univocal types with *does not equal*

**Column AF**	if you were in none of those places : *equals* *(*leave blank*)*

If you were in one of those: *equals* *(*blank*)*	**or**	*equals* *Thatplace*

***TROUBLESHOOTING***

***The plant identified does not look like the Google image*** *or* ***all rows disappeared !***

Have you respected the rules “*minima-less than-greater*” and “*maxima-greater than-smaller*“?

Have you stayed loose enough on  “how much greater” and “how much smaller” ?

Have you added for the columns that need it: ‘OR *does not equal* *(leaving blank)*’ ?
