# Supplementary material for: Legumes of the Sardinia Island: Knowledge on Symbiotic and Endophytic Bacteria and Interactive Software Tool for Plant Species Determination
Source: Plants (Basel). 2022 Jun 6;11(11):1521. doi: 10.3390/plants11111521 (PMC9183093; doi:10.3390/plants11111521)
Supplement: Supplementary file 1 [file plants-11-01521-s001.zip › Document S2. Istruzioni versione Italiana per Botabase Keys.doc]

**B O T A B A S E KEYS**

**B o t a n i c D a t a b a s e w i t h B u i l t–I n	K e y s**

**Programma a Chiavi Sottrattive per la Determinazione delle Specie Vegetali**

**Idea e Realizzazione : Andrea Squartini, Marzo 2004**

**Versione: Leguminose Sardegna**

**Nota:** la versione del file allegata è relativa a dati basati sulla prima versione di Flora d’Italia ma le ulteriori specie possono essere inserite aggiungendo righe e dati corrispondenti nelle opportune colonne.

Il file Excel di riferimento è in formato .xls e può essere utilizzato anche con versioni di Excel 1997-2003. Nelle istruzioni sono indicate sia le scelte per l’uso di tale versione, che quelle per le versioni avanzate di Excel (.xlsx)

**Uso del Filtro**

Aprire la cartella Excel “Versione italiana Botabase keys per Leguminose Sardegna.xls”, che contiene un elenco di tutte le 189 specie di Leguminose reperibili in territorio regionale Sardo, e i relativi dati morfologici e ed ecologici (Pignatti, 1982, Flora d’Italia, vol. I). I nomi di genere e specie (e i sinonimi tra parentesi) sono riportati nella colonna A.

Dalla barra di menu selezionare : Dati 🡪Filtro 🡪 Filtro automatico

appaiono in cima ad ogni colonna le frecce di altrettante finestre a tendina dalle quali si imposteranno i filtri excel (*per rimuovere il filtro : Dati 🡪Filtro 🡪 Mostra tutto*).

**Forma** (colonna B)

A : annua, B : bienne, P : perenne, PL : perenne legnosa (arbusto), PLF, perenne legnosa con fusto (albero)

Se l’esemplare che stiamo classificando è una pianta **arborea**

ovvero chiaramente lignificata, possiamo escludere tutte le piante erbacee in questo modo :

🡪Filtri per testo > Filtro Personalizzato (Excel .xlsx versions) or 🡪personalizza (Excel 1997-2003)

Mostra percorso righe:

forma

*uguale a* PL

o

*uguale a* PL,PLF

(osservare la barra in basso, indica che abbiamo ridotto l’elenco da 189 a 26 !)

Se invece è una pianta **erbacea**

escludiamo tutte le legnose

🡪Filtri per testo > Filtro Personalizzato (Excel .xlsx versions) or 🡪personalizza (Excel 1997-2003)

Mostra percorso righe:

forma

*diverso da* PL

e

*diverso da* PL,PLF

Nota 1: è importante nel primo esempio (ricerca di arborea) in cui i criteri sono “uguale a”, che la congiunzione sia “o” (non “e” !) altrimenti il programma cercherà celle in cui trovi sia PL che PL,PLF, ripetuto nella stessa cella ma naturalmente non ne esiste nessuna.

Invece nel secondo caso (ricerca di erbacea) in cui i criteri sono “diverso da”la congiunzione deve essere “e”, (“non “o”). Infatti quando diciamo “diverso da A” escludiamo A, ma se aggiungessimo “oppure diverso da B” riammetteremmo di nuovo A e di conseguenza non scarteremmo nessuno.

**Altitudine minima** (Colonna C)

Supponiamo di aver trovato la pianta in una località situata, ad esempio a circa 137 metri sul livello del mare, (valore leggibile da cartine geografiche o topografiche, o con lettore GPS). Non è comunque indispensabile sapere con esattezza l’altitudine del punto di reperimento, basterà anche una approssimazione, supponiamo quindi di poter dedurre dalle cartine che siamo tra 100 e 150 m.

col filtro vogliamo escludere tutte le piante la cui altitudine minima sia superiore a quella dove abbiamo trovato la nostra (ad es. piante di montagna che mai si troveranno sotto a una certa quota )

🡪Filtri per testo > Filtro Personalizzato (Excel .xlsx versions) or 🡪personalizza (Excel 1997-2003)

Mostra percorso righe:

altitud.min.

*minore di* 150

Nota 2: anche se teoricamente questo dovrebbe escludere le piante che non possono essere la nostra, cioè tutte quelle che mai scendono sotto i 150, in realtà in biologia i comportamenti e le forme sono piuttosto elastici, è quindi meglio, in questa e in tutte le altre filtrazioni di caratteri variabili in maniera quantitativa continua (altezza, epoca di fioritura, dimensioni fiorali, numero di foglioline etc., **tenersi un po' più larghi** e scrivere per sicurezza non 150 ma, almeno, 300 o 400. Infatti, per quanto rigoroso sia il Pignatti, è possibile che la pianta descritta come mai sinora reperita ad altitudini inferiori a tot., possa occasionalmente trovarvisi, nel qual caso, stando troppo vicini al valore da noi misurato, avremmo filtrato via la nostra dall’elenco. Tra l’altro la maggior parte dei dati di altitudine del Pignatti stesso sono arrotondati al centinaio.

Perciò in base a quanto esposto alla nota 2, è bene mettere :

🡪Filtri per testo > Filtro Personalizzato (Excel .xlsx versions) or 🡪personalizza (Excel 1997-2003)

Mostra percorso righe:

altitud.min.

*minore di* 300

**Altitudine massima** (Colonna D)

Qui vogliamo invece escludere tutte le specie che mai si spingeranno all’altitudine della nostra, nel caso dell’esempio, avendo reperito la pianta ad una quota piuttosto bassa, in pratica elimineremo le specie litoranee di spiaggia. Mentre se fossimo in alta montagna la manovra scremerebbe drasticamente l’elenco facendo sparire tutte le piante non montane.

🡪Filtri per testo > Filtro Personalizzato (Excel .xlsx versions) or 🡪personalizza (Excel 1997-2003)

Mostra percorso righe:

altitud.max.

*maggiore di* 50

Ci siamo tenuti larghi (in questo caso naturalmente al di sotto), come consigliato dalla Nota 2.

Nota 3. Per meglio ricordare come impostare i filtri su caratteri di minimi e di massimi (altitudine, altezza pianta, epoche di fioritura, lunghezza corolla, etc.) è possibile tenere a mente la seguente regola :

se la colonna riguarda *i minimi* impostare *minore* *di* e poi scrivere un valore *maggiore* di quello da noi osservato

se la colonna riguarda *i massimi* impostare *maggiore* *di* e poi scrivere un valore *minore* di quello da noi osservato.

Quindi per colonne dei minimi, quali “altitudine minima”, “altezza minima”, “fioritura a partire dal mese di” etc.

ricordarsi la sequenza mnemonica “***minimi-minore-maggiore***”

mentre per le colonne dei massimi quali “altitudine massima”, “altezza massima”, “fioritura fino al mese di” etc.

ricordarsi la sequenza mnemonica “***massimi-maggiore-minore***”

**Altezza minima** (Colonna E)

Esempio, la pianta è alta 40 cm, teniamo conto sempre della legge del largheggiare esposta all nota 2.

🡪Filtri per testo > Filtro Personalizzato (Excel .xlsx versions) or 🡪personalizza (Excel 1997-2003)

Mostra percorso righe:

altiezza.min.

*minore di* 60

(è una colonna di *minimi*, il criterio è quindi *minore di*, e scrivo un valore *maggiore di quello osservato* , cioè avendo osservato 40, scrivo, almeno, 60).

**Altezza massima** (Colonna F)

🡪Filtri per testo > Filtro Personalizzato (Excel .xlsx versions) or 🡪personalizza (Excel 1997-2003)

Mostra percorso righe:

altezza max.

*maggiore di* 20

(è una colonna di *massimi*, il criterio è quindi *maggiore di*, e scrivo un valore *minore di quello osservato* , cioè avendo osservato 40, scrivo, al più, 20).

**Fiorita da**  (Colonna G)

I numeri sono quelli del mese (3= marzo, 4=aprile etc.)

Supponiamo che la nostra pianta sia stata da noi trovata fiorita il 7 di maggio , qui vogliamo escludere le piante che inizino a fiorire solo in date posteriori. Per la regola della elasticità esposta alla nota 2, anche qui è bene non escludere di netto tutte quelle che sono riportate fiorire da giugno, meglio largheggiare di un mese, e escludere quelle dichiarate iniziare a fiorire da luglio. È infatti importante tener conto delle possibili anticipazioni o ritardi della stagione che ogni anno possono indurre a seconda dell’andamento climatico, notevoli deviazioni dalle regole fenologiche. Inoltre i valori del Pignatti sono, per molte delle specie riferiti al territorio nazionale e nella regione mediterranea vanno intesi come più tendenti ad una anticipazione.

Quindi :

🡪Filtri per testo > Filtro Personalizzato (Excel .xlsx versions) or 🡪personalizza (Excel 1997-2003)

Mostra percorso righe:

fiorita da.

*minore di* 7

**Fiorita fino a**  (Colonna H)

Qui invece vogliamo escludere tutte le piante che a maggio siano ormai sicuramente sfiorite. Come detto sopra, anziché tagliare ad aprile, mettiamo prudenzialmente marzo

🡪Filtri per testo > Filtro Personalizzato (Excel .xlsx versions) or 🡪personalizza (Excel 1997-2003)

Mostra percorso righe:

fiorita fino a.

*maggiore di* 3

**Foglie** (Colonna I)

In questa colonna i caratteri non sono quantitativi ma qualitativi, non dovremo quindi utilizzare operatori di confronto del genere maggiore o minore, bensì cercare *parole* corrispondenti al tipo osservato. La funzione di ricerca testo permette anche di cercare parti di esso, (anche solo *porzioni di parole o sillabe*), per questo se ho una foglia a tre foglioline basterà anche cercare *trif* o *ogliol* etc. Questo rende molto versatile la ricerca e permette l’uso di descrizioni anche complesse nelle celle di testo.

Innanzitutto è possibile vedere se la pianta ha foglie con cirri e viticci terminali alle foglie, nel qual caso conviene, senz’altro guardare impostare :

🡪Filtri per testo > Filtro Personalizzato (Excel .xlsx versions) or 🡪personalizza (Excel 1997-2003)

Mostra percorso righe:

foglie

*contiene*	*cir*

se inoltre si vede che la pianta ha anche fusto alato (membrane longitudinali piatte lungo il fusto) impostare :

🡪Filtri per testo > Filtro Personalizzato (Excel .xlsx versions) or 🡪personalizza (Excel 1997-2003)

Mostra percorso righe:

foglie

*contiene*	*cir*

e

*contiene* *ala*

se invece ha solo fusto alato e non ha cirri :

🡪Filtri per testo > Filtro Personalizzato (Excel .xlsx versions) or 🡪personalizza (Excel 1997-2003)

Mostra percorso righe:

foglie

*contiene* *ala*

e

*non contiene*	*cir*

(In questo caso si è già arrivati alla specie, dato che l’unica con fusto alato e senza cirri è il *Lathyrus nissolia*)

Se invece come la maggioranza delle specie la pianta non ha nè cirri nè fusto alato, osservare le foglie.

Un primo tipo facile da riconoscere è il caso delle foglie palmate, sono composte da più foglioline ma attaccate tutte nello stesso punto (come le fronde di una palma). Se presenti selezionare subito *contiene* **palmate**.

Se le foglie non sono palmate, possono essere semplici o composte da più foglioline (= segmenti) disposte a schiera come nelle penne degli uccelli (pennate), sui lati di un asse che è il prolungamento del picciolo. Il numero di foglioline quindi varia a seconda di quante serie ne porta l’asse. Ogni serie sono una coppia di foglioline e alla punta può essercene o meno una terminale spaiata (in alcune specie sostituita dai cirri). Quindi il numero di foglioline può essere pari o dispari.

Per scegliere basterà contare le foglioline. I numeri del seguente elenco corrispondono in pratica anche al numero di foglioline osservate. Il termine in grassetto è la parola da immettere nel filtro dicendo *contiene* :

1) **Semplici** : foglia non composta da foglioline, (intera)
2) **1pennate** (foglia in cui sono disposte “a penna” un solo paio cioè 2 foglioline)
3) **trifoliate :** foglia composta da tre foglioline (come nei trifogli)

3bis) **trifoliate con segmento centrale picciolettato (**le tre foglioline non sono attaccate nello stesso punto, la centrale ha un breve picciolo proprio)

4) **2pennate** (foglia in cui sono disposte “a penna” due paia, cioè 4 foglioline)
5) **5** cinque foglioline (“a lucertola”, in pratica come le 2pennate ma con fogliolina terminale

5bis) **3+2,** sempre cinque foglioline ma le due di base sono visibilmente un po' diverse essendo in realtà le stipole basali ingrandite.

>5) **>5foliate,** quando le foglioline sono più di 5, quindi la fg è almeno tri-pennata (6 o 6+1foglioline) o o quadri-pennata (8 o 8+1 foglioline) etc. etc.

Nel caso si osservino sulla stessa pianta uno o più dei vari tipi di foglie descritti, basterà impostare *contiene* ***mist*** (misto o miste)

Se infine si nota che le foglie non appartengono a nessuno dei tipi descritti e sono di forma “strana”

basterà impostare *contiene* **particolari**

Nel caso di foglie trifoliate del cui segmento centrale non si sia certi se è sessile o picciolettato perchè l’eventuale peduncolo appare presente ma quasi nullo, limitarsi a dire contiene *trif*. Questo non esclude le picciolettate e include tutte le trifoliate.

Esempio

la pianta, che non aveva cirri nè fusto alato, ha foglie composte da 12 paia di foglioline

🡪Filtri per testo > Filtro Personalizzato (Excel .xlsx versions) or 🡪personalizza (Excel 1997-2003)

Mostra percorso righe:

foglie

*contiene ****>5foli***

e

*non contiene*	*cir*

Nota 4. Attenzione nello scrivere i termini di testo come **1pennate (etc.)** senza spazio tra 1 e pennate, perché così sono scritti nelle celle, altrimenti il filtro non li trova.

**Numero minimo foglioline** (colonna J)

Quando si siano scelte le foglie >5foliate o quelle con cirri, si avranno a disposizione le due colonne J e K, in cui impostare valori contando le foglioline. (se invece si sono scelte foglie degli altri tipi il conto era già noto e si può passare alle colonne successive).

Se ad esempio, la nostra pianta ha foglie con 16 foglioline

🡪Filtri per testo > Filtro Personalizzato (Excel .xlsx versions) or 🡪personalizza (Excel 1997-2003)

Mostra percorso righe:

numero minimo di foglioline

*minore di* 18

(anche qui per la teoria del largheggiare meglio dire 18 che 16)

**Numero massimo foglioline** (colonna K)

sempre in base all’esempio a 16 foglioline

🡪Filtri per testo > Filtro Personalizzato (Excel .xlsx versions) or 🡪personalizza (Excel 1997-2003)

Mostra percorso righe:

numero massimo di foglioline

*maggiore di* 14

**Forma foglioline** (o forma foglie per le specie a foglie intere) (colonna L)

Colonna descrittiva, non predisposta per filtrazioni ma utile per verifica. Non preoccuparsi dei numerosi termini usati e passare a filtrare le 4 colonne successive.

IMPORTANTE : nelle 4 colonne su larghezza e lunghezza delle foglioline non tutte le celle sono riempite poiché per alcune specie i testi non riportano i valori, non è un problema purché si adotti l’accorgimento aggiuntivo di selezionare, oltre alle celle che soddisfano il criterio di dimensione impostato, ANCHE LE CELLE VUOTE, Basterà inserire “o” *uguale a*	e non scrivere niente nella seconda finestra di scelta. Questo significa “uguale a celle vuote”.

Mettiamo di avere una pianta le cui foglioline, (misurandone un certo numero, e naturalmente in foglie mature e completamente sviluppate) sono larghe mai meno di 3 mm e mai più di 6 mm, e sono lunghe mai meno di 10 mm e mai più di 13 mm.

**Larghezza minima foglioline** (o foglie, per le specie a foglie intere) (colonna M)

🡪Filtri per testo > Filtro Personalizzato (Excel .xlsx versions) or 🡪personalizza (Excel 1997-2003)

Mostra percorso righe:

larghezza minima foglioline

*minore di* 5

o

*uguale a*

Importante : usare o (e non e) e lasciare vuoto il campo dopo *uguale a*, solo in questo modo non saranno scartate le celle vuote.

**Larghezza massima foglioline** (o foglie, per le specie a foglie intere) (colonna N)

🡪Filtri per testo > Filtro Personalizzato (Excel .xlsx versions) or 🡪personalizza (Excel 1997-2003)

Mostra percorso righe:

larghezza massima foglioline

*maggiore di* 4

o

*uguale a*

**Lunghezza minima foglioline** (o foglie, per le specie a foglie intere) (colonna O)

🡪Filtri per testo > Filtro Personalizzato (Excel .xlsx versions) or 🡪personalizza (Excel 1997-2003)

Mostra percorso righe:

lunghezza minima foglioline

*minore di* 12

o

*uguale a*

**Lunghezza massima foglioline** (o foglie, per le specie a foglie intere) (colonna P)

🡪Filtri per testo > Filtro Personalizzato (Excel .xlsx versions) or 🡪personalizza (Excel 1997-2003)

Mostra percorso righe:

lunghezza massima foglioline

*maggiore di* 11

o

*uguale a*

È cruciale ricordarsi di scrivere anche uguale a (e lasciare vuoto il campo) altrimenti, filtrando via le celle vuote rischiamo di escludere piante che potrebbero essere la nostra ma di cui non erano noti i valori minimi e massimi di quel parametro. Come pro-memoria aggiuntivo, le intestazioni delle colonne in cui bisogna ricordarsi il fatto sono scritte in rosso.

**Infiorescenza** (colonna Q)

Distinzioni da fare : inizialmente, osservare se ci sono fiori isolati, in quel caso :

Fiori singoli, isolati

🡪Filtri per testo > Filtro Personalizzato (Excel .xlsx versions) or 🡪personalizza (Excel 1997-2003)

Mostra percorso righe:

infiorescenze

*contiene* *isolat*

e

*contiene* *1-*

in questo modo si selezionano tutti i casi in cui i fiori sono isolati, o possono esservi sia fiori isolati che fiori non isolati.

Se invece non si notano fiori isolati si sceglierà, in base a un altro principio, il tipo di infiorescenza. Sono possibili tre forme generali: il **racemo** (“a grappolo d’uva”) in cui c’è asse più o meno allungato su cui si inseriscono i fiori, (a volte numerosi, ma potrebbero essere anche due soltanto); il **capolino (“**a pon-pon” o “a margherita”**)** in cui i fiori sono innestati intorno a uno stesso punto in cima all’asse. (Anche se i fiori così inseriti fossero solo 2 si definirà ugualmente capolino se sono attaccati in uno stesso punto, mentre nel racemo sono attaccati lungo l’asse in punti diversi.) ; i **fascetti**, in cui i fiori, a gruppi (o anche “a uno solo” per fiori isolati) non sono portati da un’ infiorescenza con asse, ma sono portati direttamente sul fusto in maniera sessile (senza peduncolo) o peduncolati.

Per cui in caso di racemi :

🡪Filtri per testo > Filtro Personalizzato (Excel .xlsx versions) or 🡪personalizza (Excel 1997-2003)

Mostra percorso righe:

infiorescenze

*contiene* *racem*

mentre in caso di capolini

🡪Filtri per testo > Filtro Personalizzato (Excel .xlsx versions) or 🡪personalizza (Excel 1997-2003)

Mostra percorso righe:

infiorescenze

*contiene* *capolin*

infine se il caso è fascetti o fiori isolati sessili o peduncolati

🡪Filtri per testo > Filtro Personalizzato (Excel .xlsx versions) or 🡪personalizza (Excel 1997-2003)

Mostra percorso righe:

infiorescenze

*contiene* *sess*

e

*contiene* *fasc*

**Numero minimo fiori per infiorescenza** (colonna R)

Anche se non tutte le celle di questa e della prossima colonna contengono dati, in quanto non è citato il numero preciso, questo non rappresenta un problema per la filtrazione, in quanto, come già spiegato per le colonne M,N,O,P, basterà aggiungere al criterio di filtro anche le celle vuote in modo da non scartare casi che potrebbero corrispondere al numero di fiori per infiorescenza presente sulla nostra pianta.

Mettiamo di aver contato in varie infiorescenze della nostra pianta i fiori e che il minimo sia 12 e il massimo 20.

🡪Filtri per testo > Filtro Personalizzato (Excel .xlsx versions) or 🡪personalizza (Excel 1997-2003)

Mostra percorso righe:

n.min. fiori /infiorescenza

*minore di* 15

o

*uguale a*

**Numero massimo fiori per infiorescenza** (colonna S)

proseguendo lo stesso esempio

🡪Filtri per testo > Filtro Personalizzato (Excel .xlsx versions) or 🡪personalizza (Excel 1997-2003)

Mostra percorso righe:

n.max. fiori /infiorescenza

*maggiore di* 17

o

*uguale a*

**Dimensioni minime capolino** (Colonna T)

questa colonna serve quando si sia rimasti con trifogli, ovvero con specie che fanno fiori in capolini, che possono essere globosi o allungati, si misura qui la dimensione minima ( se sono rotondeggianti sarà uguale alla massima mentre se sono allungati è la larghezza). Poniamo di avere un capolino largo 15 e lungo 30

IMPORTANTE : per questa colonna e per la seguente, come già detto per le dimensioni delle foglioline, poiché per alcune specie i testi non riportano i valori, si deve adottare l’accorgimento aggiuntivo di selezionare, oltre alle celle che soddisfano il criterio di dimensione impostato, ANCHE LE CELLE VUOTE, Basterà inserire “o” *uguale a*	e non scrivere niente nella seconda finestra di scelta. Questo significa “uguale a celle vuote”.

🡪Filtri per testo > Filtro Personalizzato (Excel .xlsx versions) or 🡪personalizza (Excel 1997-2003)

Mostra percorso righe:

dimensioni minime capolino

*minore di* 18

o

*uguale a*

Importante : usare o (e non e) e lasciare vuoto il campo dopo *uguale a*, solo in questo modo non saranno scartate le celle vuote.

**Dimensioni massime capolino** (Colonna U)

🡪Filtri per testo > Filtro Personalizzato (Excel .xlsx versions) or 🡪personalizza (Excel 1997-2003)

Mostra percorso righe:

dimensioni minime capolino

*minore di* 26

o

*uguale a*

Anche queste due colonne hanno l’intestazione in rosso a mò di promemoria per non dimenticare di includere le celle vuote.

**Colore corolla** (colonna V)

Date le possibili soggettività e variabilità, inserire una parte della parola comune alle possibili sfumature (es. se è **rossa** scrivere **ross** che includerà così i possibili casi di rossa, rossiccia, rossastra, rosso-scura, rossa o gialla, etc. Comunque attenzione a non scrivere solo ros perchè se è rossa ma non rosea vogliamo escludere le rosa e le rosee).

Esempio :

🡪Filtri per testo > Filtro Personalizzato (Excel .xlsx versions) or 🡪personalizza (Excel 1997-2003)

Mostra percorso righe:

colore corolla

*contiene* *ross*

**Dimensioni minime corolla** (colonna W)

Supponiamo che la corolla sia lunga 10 mm (misurare la lunghezza o comunque la dimensione massima, cioè quando il vessillo è largo al punto da essere più grande della lunghezza del fiore), e teniamo sempre conto della nota 2.

Esempio :

🡪Filtri per testo > Filtro Personalizzato (Excel .xlsx versions) or 🡪personalizza (Excel 1997-2003)

Mostra percorso righe:

mm min. corolla

*è minore di* 12

**Dimensioni massime corolla** (colonna X)

Esempio :

🡪Filtri per testo > Filtro Personalizzato (Excel .xlsx versions) or 🡪personalizza (Excel 1997-2003)

Mostra percorso righe:

mm max. corolla

*è maggiore di* 8

**Altre note** (colonna Y)

Questa non è necessariamente una colonna da sottoporre a filtro, ma può fornire utili informazioni accessorie da leggere direttamente.

**Legume** (colonna Z)

Questa non è necessariamente una colonna da sottoporre a filtro, ma può fornire utili informazioni accessorie da leggere direttamente. Inoltre si presume che non sempre la pianta sarà stata reperita in stato di fruttificazione matura e che quindi potrebbe non esservi legume osservabile. Se però si dispone del legume sono chiaramente riconoscibili alcuni tipi, quello a spirale delle Medicago, (personalizza, *contiene* *spiral*), quelli ad articoli di *Hedysarum* o *Coronilla* (*contiene* *artic*), quelli con apparenti “fori” di alcune delle *Hippocrepis* (contiene “fori”), o quelli con ali del *Tetragonolobus* (*contiene*  *ali*). Per altre caratteristiche, non filtrare ma limitarsi a leggere le informazioni per verifica e confronto.

**Habitat** (colonna AA)

Utile per verificare il risultato. L’uso del filtro è possibile solo se : (1) la cella riporta un habitat univoco e (2) la nostra pianta **non è** stata trovata in un habitat appartenente a quel tipo. Si può in quel caso scartare quell’habitat. Esempio ho trovato la pianta in un campo, potrò scartare quindi quelle che crescono solo nei boschi. Per le piante il cui habitat è solo di un certo tipo (habitat univoco) si è evidenziata la cella con un colore e si è aggiunta una sillaba di codice in inglese per permettere la filtrazione in modo da evitare parole o incisi ricorrenti anche in celle ad habitat non univoco nel testo italiano. I tipi di habitat univoci codificati sono : WOODS : Boschi, etc. (inclusa macchia); SEA, presso il mare; DRY : ambienti aridi, inclusa gariga; WET : zone umide; PEAT : brughiere; CLIFF, scogliere presso il mare o rupi.

Le celle non colorate contengono più habitat possibili alla stessa pianta, e non sono quindi escludibili con il principio suddetto.

Per scartare le piante di bosco ad esempio quindi dovrò dire :

🡪Filtri per testo > Filtro Personalizzato (Excel .xlsx versions) or 🡪personalizza (Excel 1997-2003)

Mostra percorso righe:

habitat

*non contiene* WOODS

**Habitat note** (colonna AB)

Non per filtrare ma per ulteriori informazioni di verifica

**Substrato** (colonna AC) Indica, quando la dicitura è univoca (esempi : **calcare** oppure **silice)** esclusività per quel substrato. Ma naturalmente altre pianta per cui non c’è  niente di indicato potrebbero lo stesso trovarsi su uno dei due substrati. L’uso del filtro è quindi possibile solo se : (1) la cella riporta un substrato univoco e (2) la nostra pianta **non è** stata trovata su un substrato appartenente a quel tipo. Si può in quel caso scartare le piante che si trovano solo su quello. Esempio ho trovato la pianta in un luogo che so non eseere calcareo.

🡪Filtri per testo > Filtro Personalizzato (Excel .xlsx versions) or 🡪personalizza (Excel 1997-2003)

Mostra percorso righe:

substrato

*diverso da* calcare

Nota 5 qui si deve dire *diverso da* calcare, (e non, *non* *contiene **calc* o simili incisi), perché non vogliamo scartare quelle dove si sa che il substrato è generalmente calcare (gen.calcare), ma che quindi potrebbero occasionalmente essere anche su substrati non calcarei.

**Abbondanza** (colonna AD)

CC : comunissima, C : comune, R : rara, RR : rarissima

Colonna non da filtrare, è utile come informazione, e per giudicare anche da questo punto di vista l’attendibilità di identificazione.

**Endemiche** (colonna AE)

Colonna di semplice informazione, non da filtrare, i segni indicano le specie endemiche, cioèad areale circoscritto che include anche (x) o solo (**X**) la Sardegna.

**Note Geografiche** (colonna AF)

Sono riportate le indicazioni note per le specie la cui reperibilità in Sardegna è limitata alle zone indicate (in assenza di indicazioni invece è da intendersi possibile la distribuzione generalizzata).

Esempio 1, ho trovato una pianta nei dintorni di Sassari, cioè **non** riguarda nessuna delle località descritte nelle celle recanti indicazioni, posso quindi escludere le piante che sono solo lì.

🡪Filtri per testo > Filtro Personalizzato (Excel .xlsx versions) or 🡪personalizza (Excel 1997-2003)

Mostra percorso righe:

Note geografiche

*uguale a*

Cioè “uguale a vuote” Semplicemente non scrivendo niente dopo *uguale a* (oppure scegliendo direttamente **vuote** in fondo al menu a tendina, senza neanche chiedere il personalizza), La manovra restituirà solo le righe dove non c’è indicazione geografica, scartando le piante esclusivamente di quei posti.

Esempio 2. Ho trovato una pianta vicino a Cagliari

🡪Filtri per testo > Filtro Personalizzato (Excel .xlsx versions) or 🡪personalizza (Excel 1997-2003)

Mostra percorso righe:

Note geografiche

*uguale a*

o

*uguale a* *cagl.*

**DETERMINAZIONE**

Arrivati alla fine delle chiavi disponibili, saranno rimaste teoricamente da **una** a **poche** righe ancora non sottratte, se ce ne è una sola questa dovrebbe essere la pianta in nostro possesso, se ce ne sono più di una, la nostra dovrebbe essere tra di esse e per dirimere le incertezze rimaste si potranno eseguire le seguenti operazioni :

1) leggere le varie celle delle righe rimaste, specie quelle relative a forma foglie, altre note, legume, etc. per identificare anche se non filtrabili elementi che distinguano le specie rimaste in ballo (in questo senso il programma è implementabile anche con nuove differenze che emergano dalla pratica, e che una volta notate come distintive di specie possono venir aggiunte).

2) Inserire i nomi dei possibili taxa in Google o altri motori di ricerca in un browser web e cercare immagini per verificare la migliore compatibilità.

**PRO-MEMORIA OPERATIVO**

**Colonna B**	se è erbacea	*diverso da* PL	**e**	*diverso da* PL,PLF

se è legnosa	*uguale a* PL	o	*uguale a* PL,PLF

**Colonne C,E,G,J,W	(minimi)**	*minore di* ... un valore maggiore del nostro

**Colonne M,O,R,T**	**(minimi)** *minore di* ... un valore maggiore del nostro

**o**	*uguale a ...	(lasciare vuoto)*

**Colonne D,F,H,K,X**	(**massimi**)	*maggiore di*	...un valore minore del nostro

**Colonne N,P,S,U**	(**massimi**)	*maggiore di*	...un valore minore del nostro

**o**	*uguale a ...	(lasciare vuoto)*

**Colonna I**	se ha foglie con cirri	*contiene*	*cir*

se ha cirri e fusto alato	*contiene*	*cir*	e	*contiene* *ala*

se ha fusto alato e non ha cirri *contiene* *ala*	e	*non contiene*	*cir*

se non ha nè cirri nè fusto alato scegliere una tra la seguenti : *sempli*, *1penna*,*trifoli*,*picciolett*, *2penna*, *5penna*,*3+2*, *>5foli*, *palma*, *mist*, *particolar*

*contiene*  *(l’inciso scelto)*	e	*non contiene*	*cir*

**Colonna Q**	se ha fiori isolati	*contiene* *isolat*	e	*contiene* *1-*

se non vedo fiori isolati	scegliere una tra le tre possibilità

*contiene* *racem*

*contiene* *capolin*

*contiene* *sess*	e	*contiene* *fasc*

**Colonna V**	contiene *(inciso del colore corolla osservato, es : “ross”)*

**Colonna Z**	se disponibile legume dei tipi *spiral*, *artic*, *fori* o *ali* *contiene* (uno di quelli)

**Colonne AA, AC**	se escludibile tirare via un paio di tipi univoci con *diverso da*

**Colonna AF**	se non sei in nessuno dei posti particolari : *uguale a* *(*lascia vuoto*)*

se sei in uno dei posti	: *uguale a* *(lascia vuoto)*	**o**	uguale a *Quelposto*

***TROUBLESHOOTING***

***La pianta determinata non somiglia a quelle delle immagini risultanti da una verifica con motori di ricerca,*** oppure ***sono sparite tutte!***

Hai rispettato le regole minimo-minore-maggiore e massimo-maggiore-minore ?

Hai largheggiato opportunamente sul “di quanto maggiore” e “di quanto minore” ?

Hai aggiunto, per le colonne che lo richiedono : ‘o *diverso da* *(lasciando vuoto)* ?
